# Educational level and gender are associated with emotional well-being in a cohort of Dutch dialysis patients

**DOI:** 10.1186/s12882-024-03617-8

**Published:** 2024-05-22

**Authors:** Wisanne M. Bakker, Maurice Theunissen, Elife Öztürk, Elisabeth Litjens, Annemie Courtens, Marieke H. J. van den Beuken- van Everdingen, Marc H. Hemmelder

**Affiliations:** 1 Department of Internal Medicine, Maastricht University Medical Center+, P. Debyelaan 25, Maastricht, 6229 HX the Netherlands; 2 Center of Expertise for Palliative Care, Maastricht University Medical Center+, (MUMC+), Maastricht, the Netherlands; 3 CARIM Cardiovascular Research Institute Maastricht, University Maastricht, Maastricht, the Netherlands

**Keywords:** Dialysis, Educational level, Health-related quality of life

## Abstract

**Background:**

Patients undergoing dialysis have an impaired health-related quality of life (HRQOL). There are conflicting data from small series on whether patient-related factors such as educational level have an impact on experienced HRQOL. The aim of this study was to investigate the association between educational level and HRQOL in dialysis patients.

**Methods:**

In a single-center retrospective cross-sectional study HRQOL was measured using the Kidney Disease Quality of Life Short Form-36 (KDQOL-SF36) in prevalent chronic dialysis patients. Educational level was categorized into low, intermediate and high subgroups. Univariate and multivariate regression analyses were performed to assess the effects of age, gender, ethnicity, and dialysis vintage on the association between HRQOL and educational level.

**Results:**

One hundred twenty-nine chronic dialysis patients were included. Patients with an intermediate educational level had significantly higher odds of a higher emotional well-being than patients with a low educational level 4.37 (1.-89–10.13).

A similar trend was found for a high educational level (OR 4.13 (1.04–16.42), *p* = 0.044) The odds for women compared to men were 2.83 (1.32–6.06) for better general health and 2.59 (1.15–5,84) for emotional well-being. There was no interaction between gender and educational level for both subdomains. Each year of increasing age significantly decreased physical functioning (OR 0.94 (0.91–0.97)).

**Conclusions:**

Educational level and sex were associated with emotional well-being, since patients with intermediate and high educational level and females had better emotional well-being in comparison to patients with low educational level and males. Physical functioning decreased with increasing age.

**Supplementary Information:**

The online version contains supplementary material available at 10.1186/s12882-024-03617-8.

## Introduction

The prevalence of patients with chronic kidney disease (CKD) worldwide is estimated to be approximately 8–16% [1]. In 2020 the Chronic Kidney Disease Collaboration reported that the number of patients who were treated with kidney replacement therapy (KRT) has reached 2.5 million worldwide [2]. CKD and dialysis in particular are associated with a high mortality and morbidity, as well as impaired health-related quality of life (HRQOL) and increased disease burden due to disease specific symptoms, dialysis dependency, restricted food and drink intake, medication need and adverse effects and a reduced physical capacity [3].

To measure health-related quality of life in patients with CKD the International Consortium for Health Outcome Measurement (ICHOM) recently identified Patient-Reported Outcome Measures (PROMS) that are suitable to qualify HRQOL. Three recommended instruments are the 36-Item Short Form Health Survey (SF-36) version 2, the combination of the Patient-Reported Outcomes measurement Information System (PROMIS)- Global Health/ -29 and the Research and Development-36 (RAND-36) [4]. Important domains in these PROMS are general HRQOL, physical function, depression, pain, fatigue, and daily activity [4]. In addition, disease specific PROMS have been developed, such as the Kidney Disease Quality of Life Short Form-36 (KDQOL-SF36), which is recommended by experts of the European Renal Association (ERA) [5].

Health-related quality of life (HRQOL) has been extensively studied in dialysis patients and several patient-related factors have been identified that may affect both clinical outcomes and perceived HRQOL. To date, low educational level was shown to be associated with lower health outcomes in both the general population and dialysis patients [6–9]. However, there are inconclusive results on the association between educational level and HRQOL in dialysis patients. Data from Taiwanese, Chinese and Saudi-Arabian populations of dialysis patients showed no significant association between educational level and HRQOL [10–12]. A few studies did find an association between educational level and HRQOL. Seica et al. showed a negative association between educational level and HRQOL in a cohort of dialysis patients in Romania [13]. In contrast, two studies found a positive association between educational level and HRQOL in dialysis patients [14, 15]. In a cohort of Chinese dialysis patients, higher educational levels were associated with higher scores on the mental component of the SF-36 [11]. Daniel et al. observed more depressive symptoms in dialysis patients with a low educational level compared to dialysis patients with a higher educational level, although a lower experienced HRQOL was observed in patients who were higher educated [16]. Considering these discrepant results in dialysis populations from different regions, the aim of this study was to investigate the association between educational level and HRQOL focusing on the KDQOL-SF36 subscales emotional well-being, physical functioning, general health and pain in a Dutch cohort of dialysis patients. Secondary aim was to examine the effects of age, gender, ethnicity, and dialysis vintage on the association between the KDQOL-SF36 subscales and educational level.

## Methods

### Study design

A single-center retrospective cross-sectional study was conducted among patients undergoing either hemodialysis (HD) or peritoneal dialysis (PD) at the Maastricht University Medical Center in the Netherlands. KDQOL-SF36 records completed by patients between november 2015 and february 2021 were collected for data analysis. Records were excluded from analysis in case of unknown educational status of the involved patient. This study was exempted from the Human Subjects Act and approved by the medical-ethical evaluation board.

### Data collection

Once a year patients were approached by the nurse of the dialysis ward to complete a questionnaire on patients’ demographics (e.g. age, gender, ethnicity and educational level) and the KDQOL-SF36 (e.g. scores on the domains of emotional well-being, physical functioning, general health and pain). Patients filled in the questionnaires once a year in the context of regular care. After the first year numbers of follow-up were low, therefore only the first follow-up moment was used within this analysis. Additional data on dialysis vintage and dialysis modality were obtained from the medical files. Educational level was categorized into three subgroups: low, intermediate and high educational level. These subgroups were based on the classification as stated by the Dutch central office for statistics (CBS). Low educational level consists of no education, primary school, or lower vocational education; intermediate educational level consists of intermediate general or vocational secondary education, and pre-university education; high educational level consists of higher vocational education and university [17]. If the educational level was reported as ‘other’, these patients were added to ‘intermediate’ education level.

### Measuring quality of life in dialysis patients

Health related quality of life (HRQOL) in dialysis patients was measured by the Kidney Disease Quality of Life Instrument 36-items Short Form (KDQOL-SF36), which has been validated among different countries. The Dutch version of the KDQOL-SF36 showed overall a high validity, with an item-internal consistency ranging from 0.26 (for only two aspects: ‘work status’ and ‘quality of social interaction’) to 0.90 and a reliability ranging from 0.39 (the aspects mentioned before) to 0.95 [18]. The KDQOL-SF36 is a questionnaire based on self-reported health-related quality of life (HRQOL). Scores range from zero to 100, with a score of 100 indicating best possible health status. The questionnaire consists of several domains, which comprise of subjects such as general health, physical functioning, social functioning, emotional and mental health, vitality, and bodily pain [19, 20]. The outcome measures of this study were scores on the KDQOL-SF36 subscales emotional well-being, physical functioning, general health, and pain. These specific KDQOL-SF36 subscales were chosen as these appeared to be of clinical relevance and appropriate for the study question in the dialysis population.

### Statistical analysis

Data were presented as medians and interquartile ranges (IQR), or absolute numbers and percentages. The scores on the subscale’s emotional well-being, physical functioning and general health were dichotomized using the median split-procedure because of their non-parametric distribution and the absence of further data on cut-off values for these variables in literature [21]. Based on the median split procedure the following cut-off values were applied: 78 for emotional well-being, 60 for physical functioning and 37.5 for general health. Based on the literature on numeric rating scales in pain measurement where a cut-off value of 40 is commonly used (score > 40 implicates pain) [22–25], a cut-off of 60 was derived for the KDQOL-SF36 subscale pain (score < 61 implicates pain). Subgroup analyses were performed using the Mann–Whitney U test or Chi square test.

To investigate the association between educational level and the subscales of the KDQOL-SF36 (emotional well-being, physical functioning, general health and pain) univariate and multivariate logistic regression analysis were performed. Logistic regression analysis was chosen, since for all four subscales of the KDQOL the assumption of normality was not met. Educational level was added to each model and the following potential confounders were added to the multivariate models using forced entry: age, gender, and ethnicity (by proxy country of origin: the Netherlands versus other). Interaction of age, gender, ethnicity and dialysis vintage with educational level for each subdomain was also tested. Results are presented as odds ratio (OR) and 95% confidence interval (CI). Missing data were not imputed, however to investigate the representativeness of the included patients the characteristics of patients with a known educational level were compared to patients with an unknown educational level. A *p*-value < 0.05 was considered statistically significant for the final model. Analysis was executed in SPSS version 25 (IBM Inc, ® New York, USA).

Ethics approval and consent to participate, according to national guidelines and permission of the Medical Ethics Committee, was not required since participants were not subjected to procedures or required to follow rules or behavior [26]. The KDQOL-36 questionnaire was used as part of regular care. Patients gave informed consent to use their data for research purposes.

## Results

### Patient characteristics

**Fig. 1:**
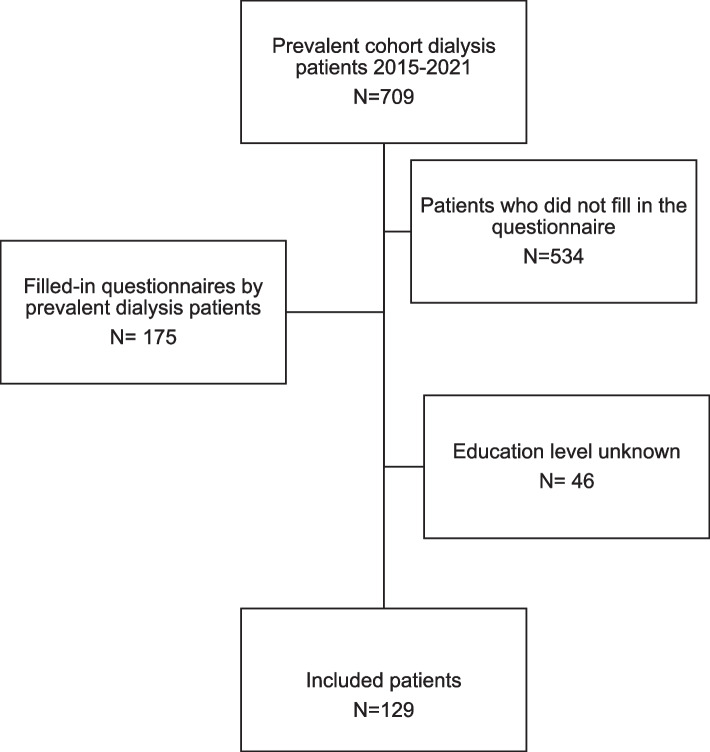
Flow chart of participant inclusion and exclusion

Out of a total of 709 prevalent dialysis patients, 175 patients completed the KDQOL-SF36 questionnaire, resulting in a response rate of 25%. Of these patients 129 were eligible for analyses in this study (Fig. 1). Baseline characteristics are presented in Table 1. The median age of the patients was 65.0 years and 58% of the patients were male. The median age did not differ between men (67.0) and women (63.0) (*p* = 0.673). Dialysis vintage ranged from 1 to 249 months with a median dialysis vintage of 15.0 months. The educational level ranged from low (41.1%), intermediate (47.3%) to high (11.6%). Concerning the KDQOL-SF36 subdomains 48.1% of the patients had a high score for emotional well-being, 51.9% of the patients had a high score on physical functioning, 45.7% patients had a high score for general health and 47.3% of the patients reported pain. Median scores of KDQOL-subscales per educational level are stated in Table 2.

**Table 1:** Patient characteristics (*n* = 129)

**Age** in years	65.0 (56.5–75.5)
Male	75 (58.1%)
**Ethnicity**	
Caucasian	104 (80.6%)
Other	21 (16.3%)
Unknown	4 (3.1%)
**Dialysis modality**	
HD	109 (84.5%)
PD	20 (15.5%)
Dialysis vintage in months	15.0 (4.6–45.2)
**Educational level**^a^	
Low	53 (41.1%)
Intermediate	61 (47.3%)
High	15 (11.6%)
**KDQOL-SF36 subdomains**	
Emotional well-being	76 (60.0–92.0)
Physical functioning	60 (32.5–80.0)
General health	35 (25.0–50.0)
Pain	65 (40.0–90.0)

Numbers represent median (interquartile range) or number (%). *Abbreviations*: *KDQOL-SF36* Kidney Disease Quality Of Life Short-Form 36, *HD* Hemodialysis, *PD* Peritoneal Dialysis

^a^*Educational level:* Low educational level: no education, primary school, or lower vocational education, intermediate educational level: intermediate general or vocational secondary education and pre-university education, high educational level: higher vocational education and university

**Table 2:** Health related quality of life and educational level

	**Educational level** **Low**	**Educational level** **Intermediate**	**Educational level** **High**	**Cut-off subscales**^a^
**Emotional well-being**				
	68.0 (56.0–82.0)	80.0 (64.0–92.0)	82.0 (64.0–96.0)	78.0
Low	37 (69.8%)	24 (39.3%)	6 (42.9%)	
High	16 (30.2%)	37 (60.7%)	8 (57.1%)	
**Physical functioning**				
	52.5 (25.0–80.0)	60.0 (32.5–85.0))	67.5 (38.8–90.0)	60.0
Low	30 (55.6%)	27 (44.3%)	5 (35.7%)	
High	24 (44.4%)	34 (55.7%)	9 (64.3%)	
**General health**				37.5
	35.0 (25.0–45.0)	40.0 (25.0–52.5)	32.5 (23.8–53.8)	
Low	31 (57.4%)	30 (49.2%)	9 (64.3%)	
High	23 (42.6%)	31 (50.8%)	5 (35.7%)	
**Pain**				
	56.5 (33.0–90.0)	68.0 (45.0–90.0)	72.5 (45.0–100.0)	60.0
Yes	30 (55.6%)	26 (42.6%)	5 (35.7%)	
No	24 (44.4%)	35 (57.4%)	9 (64.3%)	

KDQOL median (interquartile range) and numbers (%). Missing data: Emotional well-being 1

^a^Cutt-off values for the subscales emotional well-being, physical functioning and general health were derived from the median split procedure. The cut-off value for the subdomain pain was based on literature on numeric rating scales in pain measurement

The baseline characteristics of patients with an unknown educational level (*n* = 46) showed a longer dialysis vintage of median 54.5 (22.8–76.3) versus 15.0 (IQR 4.6–45.2) months in comparison to patients with a known educational level (Table S1; *p* =  < 0.001). Patients with an unknown educational level were also significantly older than patients with a known educational level (72.5 (54.5–80.3) versus 65.0 (56.5–75.5) years, *p* = 0.047). The scores on the KDQOL-SF36 subscales general health (25.0 (15.0–45.0)) versus 35.0 (25.0–50.0), *p* = 0.018) and physical function (40.0 (10.0–68.8) versus 60.0 (32.5–80.0), *p* = 0.014) were lower for patients with a unknown versus a known educational level.

### Univariate and multivariate logistic regression analysis

#### Emotional well-being

Educational level was significantly associated with emotional well-being (Table 3). Univariate analysis showed that patients with an intermediate educational level had significantly higher odds of higher emotional well-being compared to patients with a low educational level (OR 3.57 (1.64–7.77) *p* = 0.001). This was confirmed in the multivariate analysis (OR 4.37 (1.89–10.13), *p* < 0.001; Table 3). A similar trend was found for patients with a high educational level in both the univariate analysis (OR 3.08 (0.91–10.3)*p* = 0.068) and the multivariate analysis (OR 4.13 (1.04–16.42),*p* = 0.044. In addition, the odds of higher emotional well-being for women were 2.59 (1.15–5.84) compared to men, *p* = 0.022. There was no interaction between educational level and gender, age, ethnicity or dialysis vintage for this subdomain. Neither age, ethnicity, nor dialysis vintage had an significant effect on emotional well-being for any of the educational levels.

**Table 3:** Multivariable regression model

Variables	KDQOL-SF36 subdomains			
	**Emotional well-being**	**Physical functioning**	**General health**	**Pain**
Intercept	0.43	44.1	0.38	0.70
**Educational level**^a^				
Low	Reference	Reference	Reference	Reference
Intermediate	4.37 (1.89–10.13)	1.66 (0.74- 3.76)	1.55 (0.72–3.37)	0.61 (0.28–1.30)
High	4.13 (1.04–16.42)	1.60 (0.40–6.40)	0.73 (0.20–2.73)	0.52 (0.14–2.00)
**Gender**				
Male	Reference	Reference	Reference	Reference
Female	2.59 (1.15–5.84)	1.75 (0.78–3.90)	2.83 (1.32–6.06)	0.50 (0.24–1.05)
**Age (years)**	0.99 (0.97–1.02)	0.94 (0.91–0.97)	1.0 (0.98–1.03)	1.01 (0.99- 1.04)
**Ethnicity**^**b**^				
Dutch	Reference	Reference	Reference	Reference
Other	0.50 (0.17–1.47)	0.48 (0.16–1.42)	0.80 (0.29- 2.16)	1.16 (0.43–3.10)
**Nagelkerke R square**	0.20	0.22	0.09	0.07

*N* = 129. Numbers represent Odds ratio (95% confidence interval). *Abbreviations*: *KDQOL-SF36* Kidney Disease Quality Of Life Short-Form 36

^a^*Educational level:* Low educational level: no education, primary school, or lower vocational education, intermediate educational level: intermediate general or vocational secondary education and pre-university education, high educational level: higher vocational education and university

^b^*Ethnicity*: Country of origin

#### Physical functioning

Educational level was not associated with the subdomain physical functioning in both the univariate analysis (intermediate (OR 1.57 (0.75- 3.29), *p* > 0,05) and (high (OR 2.25 (0.67–7.61), *p* > 0.05)) and the multivariate analysis (intermediate (OR 1.66 (0.74–3.76), *p* > 0.05) and (high (OR 1.60 (0.40 -6.40), *p* > 0.05). Only age had a significant association with physical functioning; each year that age increased lowered the odds on good physical functioning with 0.94 (0.91 – 0.97), *p* < 0.001. There was no interaction between age, gender, ethnicity or dialysis vintage and educational level for this subdomain.

#### General health

Educational level was not associated with the subdomain general health again in both the univariate analysis (intermediate OR 1.39 (0.67–2.91)), *p* > 0,05) and high OR 0.75 (0.22–2.53)), *p* > 0.05)) and the multivariate analysis (intermediate (OR 1.55 (0.72–3.37), *p* > 0.05) and (high (OR 0.73 (0.20- 2.73), *p* > 0.05)). However, gender was significantly associated with general health. Women had a 2.83 odds ratio for a higher score on the subdomain general health compared to men (1.32–6.06), *p* = 0.007. There was no interaction between age, gender, ethnicity or dialysis vintage and educational level for this subdomain.

#### Pain

Educational level was not associated with the subdomain pain. The odds in the univariate analysis were 0.59 (0.28–1.24), *p* > 0.05 versus 0.44 (0.13–1.50) *p* > 0.05 for intermediate educational level and high educational level, respectively. Similarly, no association was found in the multivariate analysis where the odds for intermediate educational level was 0.61 (0.28—1.30), *p* > 0.05 and 0.52 (0.14–2.00), *p* > 0,05 for high educational level. There was no association or interaction with age, gender, ethnicity or dialysis vintage for this subdomain.

## Discussion

Educational level was associated with emotional well-being in this Dutch cohort study of dialysis patients. Dialysis patients with an intermediate educational level have a higher emotional well-being score compared to patients with a low educational level, whereas a similar trend was found for patients with a high educational level. No association was found between educational level and the other three KDQOL-SF36 subdomains physical functioning, general health and pain.

Our findings on emotional well-being are consistent with some previous studies in dialysis patients that found an association between educational level and mental status. Germin-Petrovic et al. observed that the subdomain ‘role emotional’ of the SF-36 was positively associated with years of education [11]. Zhou et al. found that dialysis patients scored higher on the mental component of the SF-36 as they attained a higher educational level [14]. Bayoumi et al. and Rostami et al. also found that educational level was positively associated with the SF-36 domain emotional well-being [26, 27]. These differences in emotional well-being between educational levels could be due to variations in health literacy as patients with a lower level of education often have lower heath literacy, which in turn is associated with poorer health and a lower quality of life [28, 29]. Other factors that may play a role in this association are for instance socio-economic status as lower educational level often comes along with a lower socio-economic status, which may lead to financial worries affecting mental health. However, two other studies by Seica et al. and Kao et al. found no association between educational level and emotional well-being [10, 13]. This discrepant result could be due to differences in study characteristics as our study was conducted in a smaller study population. Furthermore, their study only addressed HD patients who were younger (mean age 51.7 and 59.4 years) compared to our study population (mean age 63.9 years). These differences could explain the conflicting results on emotional well-being in relation to educational level. Dialysis may play a more prominent role in the daily lives of younger patients, for example by affecting employment and socio-economic status and thereby affect perceived HRQOL.

No association was observed between educational level and the other three KDQOL-SF36 subdomains, physical functioning, general health and pain. These findings are consistent with those of Kao et al. who reported no association between educational level and these domains by using a variant of the SF-36 in a cohort of 861 Taiwanese HD patients [10]. A negative association between level of education and these KDQOL-SF36 domains was observed by Seica et al. Dialysis patients with a higher educational scored lower on these domains [13]. A positive association between educational level and these SF-36 subdomains was observed in two other studies [26, 27]. These contrasting findings may be due to differences in study population characteristics due to inclusion of only HD patients in comparison to a mixed cohort of both HD and PD patients. Previous research showed that PD patients experienced a better HRQOL than HD patients [30, 31].

We also observed that the patient characteristics age and gender were associated with the KDQOL-SF-36 subdomains physical functioning, general health and emotional well-being. Firstly, age was associated with a decline in physical functioning. This was also observed in previous studies showing that older subjects in a cohort of the Dutch general population scored lower on the SF-36 components of physical functioning compared to their younger counterparts [32]. Bayoumi et al. reported a similar finding in a cohort of 709 HD patients with a mean age of 51.7 years. A lower score in the ‘physical functioning’ domain of the SF-36 was observed in patients > 65 years of age compared to patients < 65 years of age [26]. A decrease in physical functioning with increasing age was also observed by AL-Jumaih et al. in a cohort of 100 HD patients with a mean age of 53.4 years. Patients aged > 40 years scored lower on the physical component score than patients < 40 years [12]. Carmichael et al. also observed a decrease in the experienced physical role limitation with an increasing age, particularly in women. This suggests that older patients are able to better adjust to limitations by their disease [33]. In our study no effect of gender on physical functioning was found. In comparison to Carmichael et al., our patients had a longer dialysis vintage (2.85 years versus 2.12 years) and were older (63.9 years versus 57.8 years). A longer vintage increases the time that both male and female patients have to adapt to their decline in physical functioning, possibly fading out gender differences. Secondly, gender did have a significant effect for the subdomain general health in our study. Women had a higher score compared to men, in agreement with Bayoumi et al. [26]. Rostami et al. demonstrated the opposite with male dialysis patients experiencing a better HRQOL [27, 34]. This discrepancy could be due to a difference among countries in gender status. Thirdly, we demonstrated higher emotional well-being scores in women compared to men. In contrast, man showed higher mean mental component scale (MCS) scores compared to women in a Taiwanese population of 497 HD patients. Female HD patients had higher depression scores in a population of 868 HD patients in Brazil [35]. An explanation for these discrepancies could be the differences in social status between man and women in the involved countries. No patient characteristics were associated with the subdomain pain in our study. Several other studies did find associations of these variables with pain in HD patients [36–39]. Especially, female HD patients experiencing more pain compared to men [36–38]. The discrepancy may be due to inclusion of HD patients only in these studies, as previous research showed that HD patients experience more pain due to vascular access cannulation in comparison to PD patients [30]. Moreover, pain in these studies has been assessed by specific questionnaires for pain and not the KDQOL-SF36.

Our study adds new insights to the diversity of results on the association between educational status and HRQOL in dialysis patients, as it was conducted in a European population. Previous studies were mainly conducted in Asian and Middle-east countries. In addition, we performed a validated disease specific questionnaire by the KDQOL SF-36 and examined the effects of the main patient characteristics age, gender, ethnicity and dialysis vintage on the association between educational level and HRQOL.

A limitation is that we conducted a single center study with a relatively small study population. This may reduce the generalizability of our results. Selection bias was introduced as only patients who completed the questionnaire and reported their level of education were included in the analysis. Since 25% of the patients in the centre completed the questionnaire, selection bias in our population could have affected the association. Patients that have an intrinsic motivation to fill in the questionnaire may fill in the questionnaires in another way than patients who didn’t. It could be that level of education and health literacy could have an effect as patients with a lower level of education and lower health literacy were less likely to fill in the questionnaire and that these patients were missed and thereby these results could be an underestimation. When comparing patient available characteristics of the overall dialysis population in the Maastricht UMC with the patients included in this analysis the percentage of patients undergoing hemodialysis (approximately 84% in both cohorts) and percentage of males (approximately 58% in our cohort and approximately 60% in the overall population) is quite similar. Moreover, when comparing the group with known educational level to the group with unknown educational level they did not differ on the level of emotional well-being. The lower scores on general health and physical functioning in the patients with an unknown educational level could be due to the longer dialysis vintage and higher age that has been found in these patients. Longer dialysis vintage and higher age could reduce their physical condition and thus affect their percieved general health and physical functioning [12, 32, 40]. Lastly, lacking data on occupational status, depressive symptoms and anxiety is a limitation of this study as these could provide additional insight into mediating effects on the relationship between educational level and HRQOL.

Our findings may have implications for clinical practice as educational level should be taken into account when assessing HRQOL in dialysis patients. Attention to patient’s emotional well-being is particularly important in male patients with a low educational level. To gain further insight in HRQOL in clinical practice, patient-reported-outcome measures were incorporated in Dutch dialysis care [41, 42].

In conclusion, educational level and gender were associated with emotional well-being in this dialysis cohort. Patients with intermediate and high educational level showed higher levels of emotional well-being in comparison to patients with low educational level. Men were at higher risk of poorer general health and emotional well-being than women. Healthcare professionals could provide a mental support intervention adapted to the educational level when patients report a low mental health status to improve their quality of life.

### Supplementary Information

Supplementary Material 1.

## Data Availability

The datasets used and/or analyzed during the current study are available from the corresponding author on reasonable request.

## Abbreviations

CKD: Chronic kidney disease
KRT: Kidney replacement therapy
HRQOL: Health-related quality of life
ICHOM: The International Consortium for Health Outcome Measurement
PROMS: Patient-Reported Outcome Measures
SF-36: 36-Item Short Form Health Survey
PROMIS: Patient-Reported Outcomes measurement Information System
RAND-36: Research and Development-36
KDQOL-SF36: Kidney Disease Quality of Life Short Form-36
ERA: European Renal Association
HD: Hemodialysis
PD: Peritoneal dialysis
CBS: Central office for statistics
CI: Confidence interval
OR: Odds ratio
