# Supplementary material for: Educational level and gender are associated with emotional well-being in a cohort of Dutch dialysis patients
Source: BMC Nephrol. 2024 May 22;25:179. doi: 10.1186/s12882-024-03617-8 (PMC11112868; doi:10.1186/s12882-024-03617-8)
Supplement: Supplementary file 1 — Supplementary Material 1. [file 12882_2024_3617_MOESM1_ESM.docx]

**Supplementary:**

**Table S1. Comparison patientcharacteristics patients known and unknown educational level.**

| **Educational level^a^** | **Known (n=129)** | **Unknown (n=46)** | **P-value** |
| --- | --- | --- | --- |
| **Age in years** | 65.0 (56.5-75.5) | 72.5 (54.5-80.3) | 0.047 |
| **Male** | 75 (58.1) | 24 (63.2) | 0.580 |
| **Dialysis vintage in months** | 15.0 (4.6-45.2) | 54.5 (22.8-76.3) | <0.001 |
| **Ethnicity** |  |  |  |
| Caucasian | 104 (80.6%) | 7 (18.4%) | 0.627 |
| Other | 21 (16.3%) | 0 (0.0%) |  |
| Unknown | 4 (3.1%) | 31 (81.6%) |  |
| **Dialysis modality** |  |  |  |
| HD | 109 (84.5%) | 37 (80.4%) | 0.894 |
| PD | 20 (15.5%) | 9 (19.6%) |  |
| **Mean scores KDQOL-SF36 subdomains** |  |  |  |
| Emotional well-being | 76.0 (60.0-92.0) | 76.0 (56.0-88.0) | 0.515 |
| Physical functioning | 60.0 (32.5-80.0) | 40.0 (10.0-68.8) | 0.014 |
| General health | 35.0 (25.0-50.0) | 25.0 (15.0-45.0) | 0.018 |
| Pain | 65.0 (40.0-90.0) | 58.0 (33.0-80.0) | 0.296 |

Numbers represent median (interquartile range) or number (%). Abbreviations: KDQOL-SF36, Kidney Disease Quality Of Life Short-Form 36; HD, Hemodialysis; PD, Peritoneal Dialysis

*^a^Educational level:* Low educational level: no education, primary school, or lower vocational education, intermediate educational level: intermediate general or vocational secondary education and pre-university education, high educational level: higher vocational education and university.
